# Family leadership styles and adolescent dietary and physical activity behaviors: a cross-sectional study

**DOI:** 10.1186/1479-5868-9-48

**Published:** 2012-04-30

**Authors:** Katie L Morton, Alexandra H Wilson, Lisa S Perlmutter, Mark R Beauchamp

**Affiliations:** 1 Primary Care Unit, Department of Public Health and Primary Care, Institute of Public Health, University of Cambridge, Cambridgeshire, CB2 0SR, UK; 2 School of Kinesiology, The University of British Columbia, Vancouver, V6T 1Z1, Canada

**Keywords:** Family, Leadership, Parenting, Adolescent health, Physical activity, Nutrition

## Abstract

**Background:**

Transformational leadership is conceptualized as a set of behaviors designed to inspire, energize and motivate others to achieve higher levels of functioning, and is associated with salient health-related outcomes in organizational settings. Given (a) the similarities that exist between leadership within organizational settings and parenting within families, and (b) the importance of the family environment in the promotion of adolescent health-enhancing behaviors, the purpose of this exploratory study was to examine the cross-sectional relationships between parents’ transformational leadership behaviors and adolescent dietary and physical activity behaviors.

**Methods:**

857 adolescents (aged 13–15, mean age = 14.70 yrs) completed measures of transformational parenting behaviors, healthful dietary intake and leisure-time physical activity. Regression analyses were conducted to examine relationships between family transformational leadership and adolescent health outcomes. A further ‘extreme group analysis’ was conducted by clustering families based on quartile splits. A MANCOVA (controlling for child gender) was conducted to examine differences between families displaying (a) HIGH levels of transformational parenting (consistent HIGH TP), (b) LOW levels of transformational parenting (consistent LOW TP), and (c) inconsistent levels of transformational parenting (inconsistent HIGH-LOW TP).

**Results:**

Results revealed that adolescents’ perceptions of family transformational parenting were associated with both healthy dietary intake and physical activity. Adolescents who perceived their families to display the highest levels of transformational parenting (HIGH TP group) displayed greater healthy eating and physical activity behaviors than adolescents who perceived their families to display the lowest levels of transformational parenting behaviors (LOW TP group). Adolescents who perceived their families to display inconsistent levels of transformational parenting behaviors (HIGH-LOW TP group) displayed the same levels of healthy eating behaviors as those adolescents from the LOW TP group. For physical activity behaviors, adolescents who perceived their families to display inconsistent levels of transformational parenting behaviors (HIGH-LOW TP group) did not differ in terms of physical activity than those in either the HIGH TP or LOW TP group.

**Conclusions:**

Family transformational parenting behaviors were positively associated with both healthful dietary intake and leisure-time physical activity levels amongst adolescents. The findings suggest that transformational leadership theory is a useful framework for understanding the relationship between family leadership behaviors and adolescent health outcomes.

## Background

Unhealthy lifestyles characterized by poor nutrition and sedentary behaviors are associated with a range of health problems during youth and, if continued into adulthood, may contribute to the development of chronic conditions such as cardiovascular disease, Type 2 diabetes, certain cancers and osteoporosis [1,2]. Research has demonstrated that the home environment is especially important for fostering adolescent health, as families (especially parents) represent potential role models and sources of support for numerous health behaviors [3-6].

Good leadership has long been recognized as imperative for the effective growth and development of organizations [7,8], and it has been suggested that the same is required within families in order to establish a climate and family culture that is conducive to healthy adolescent development [9,10]. In spite of the need for effective leadership in families, theory and research in this area are limited. The majority of research surrounding families and adolescent health has predominantly focused on parenting styles and parenting practices [11-14], with little attention given to the role of effective leadership behaviors displayed by parents, and how family leadership behaviors might impact upon adolescent health.

The theoretical framework utilized in the present study corresponds to transformational leadership theory [15]. Transformational leadership was originally conceptualized in organizational settings and has grown to become the most widely utilized model of leadership of the past two decades [16]. There are two reasons why this framework is pertinent to our understanding of families and adolescent health. First, transformational leadership behaviors are in many ways synonymous with effective parenting behaviors [17]. For example, both transformational leadership and parenting are concerned with influencing others towards common goals and objectives and involve inspiring and motivating others to take ownership and self-regulate their own behaviors in order to achieve these goals. Furthermore, effective parenting and transformational leadership require mutual trust, integrity and empathy to enhance the relationship quality that exists between leader and follower. It is these conceptual similarities that make transformational leadership theory a particularly viable framework for understanding the role of family leadership behaviors in the promotion of adolescent health.

Second, transformational leadership has been studied a range of organizations and settings, including the military [18,19], businesses [20,21], sport [22,23], physical education [24,25] and more recently, parenting [10,26]. This research has consistently demonstrated that transformational leadership behaviors are associated with a host of adaptive outcomes among those being led, such as greater motivation [22,24], self-efficacy [20,26,27], well-being [26,28], and achievement [21,29]. Furthermore, a growing body of experimental research has demonstrated that transformational leadership can be taught and developed through short-term intervention [30]. This highlights the important utility of extending transformational leadership to the family domain with a view to understanding and potentially fostering family transformational leadership behaviors to bring about improved adolescent health outcomes.

So what exactly is transformational leadership? This style of leadership involves behaviors that empower and inspire those being led to achieve higher levels of functioning [15]. When applied to the parenting domain [cf. [9], and consistent with transformational leadership theory [8,15] transformational parenting comprises four key behavioral dimensions namely *idealized influenceinspirational motivationintellectual stimulation* and *individualized consideration*. Idealized influence takes place when the parent behaves as a role model, through the demonstration of their own values, and includes behaviors that engender the trust and respect of their children. Inspirational motivation involves behaviors that inspire and energize their children to fulfill their potential, and whereby parents are optimistic about what their children can achieve. Intellectual stimulation occurs when parents encourage their children to think for themselves and to approach old problems in new ways. Finally, individualized consideration involves behaviors that display a genuine sense of care, concern and compassion and take into consideration children’s unique developmental needs.

In the first empirical study to extend transformational leadership theory to the parenting and adolescent health domain Morton and colleagues [26] developed a measure of transformational parenting for use with adolescents and demonstrated positive associations between adolescents’ perceptions of transformational parenting and greater self-regulatory efficacy for healthy eating and physical activity. The focus of the present study was to extend this line of research and explore associations between adolescents’ perceptions of transformational parenting behaviors that exist within families and two salient adolescent health-enhancing *behaviors*, namely healthy dietary intake and leisure-time physical activity. Indeed, a limitation of the Morton et al. [26] study was that actual health *behaviors* were not examined.

Furthermore, in this preliminary study by Morton et al., the role of individual parents’ (mothers and fathers) was explored rather than taking into consideration the influence of multiple (i.e., which is typically two) parents within the family unit. Within the leadership literature, participants are typically asked to rate the behaviors of a single leader to whom an employee directly reports [31]. Thus, little is known about the effects of having two (or more) leadership figures that may be consistent in their levels of transformational leadership (either both displaying high levels of transformational leadership behaviors or both displaying relatively low levels) or perhaps the case where one leader is highly transformational and the other displays relatively low levels of transformational leadership. Nevertheless, in the organizational psychology literature, it has been suggested that having multiple leaders operate through consistent displays of transformational leadership (i.e., both leaders displaying high levels of transformational leadership behaviors) may have a positive effect on followers’ motivation, whereby each leader’s behavior can complement or reinforce those of another [31]. In such instances the effects of each leader may be additive. Similarly, in the parenting literature, it is argued that having two *authoritative* parents is better than one, and having one is better than having none, even if it means the parents do not see eye to eye in terms of their respective parenting approaches [32]. Although research has demonstrated that within two-parent families mothers’ and fathers’ behaviors are usually moderately to highly correlated [33,34] an interesting question relates to the cases where one parent in the family unit is perceived to display high levels of transformational parenting behaviors and the other perceived as displaying low levels of transformational parenting behaviors. For example, is one parent displaying high levels of transformational parenting behaviors sufficient to bring about adaptive health-related outcomes in adolescents or are both parents (i.e., the entire family unit) required to display high levels of transformational parenting (reflecting inter-parental consistency) in order to positively predict adolescent health behaviors?

With this in mind, it was hypothesized that (a) adolescents’ perceptions of family transformational parenting behaviors would be associated with improved dietary intake and greater leisure-time physical activity and (b) that adolescents’ who perceive their families to display consistent high levels of transformational parenting behavior (consistent HIGH TP) would display more healthy eating and physical activity behaviors than those families perceived to display consistently low levels of transformational parenting (consistent LOW TP). It was further hypothesized that adolescents who perceived their families to display inconsistent levels of transformational parenting (HIGH-LOW TP) would display healthier eating and greater physical activity behaviors than families in the consistently LOW TP group, but less health enhancing behaviors than the consistently HIGH TP group.

## Methods

### Participants

Participants were 857 adolescents between 13 and 15 years (*M*_*age*_ = 14.70 yrs; 426 males, 426 females, with 5 who did not specify their gender)^1^. No other inclusion/exclusion criteria (other than aged 13–15) were specified. Participants were recruited from thirty five classes in four schools in the Lower Mainland of British Columbia (Canada), and represented a diverse range of ethnic and socioeconomic backgrounds. Specifically, 21% of adolescents identified themselves as Canadian, 27% Canadian-Asian, 40% Asian (of this 40%, the majority (28%) identified themselves as Chinese, 4% Korean, 3% Filipino, 3% Vietnamese and 2% ‘other’), 5% East Indian and 7% ‘other ethnicities’. The composition of the sample was representative of the ethnic composition of this area of Canada.

### Procedures

Ethical approval was obtained from the Research Ethics Board at the University of British Columbia, along with School Board approval. Potential schools were contacted and once schools had elected to participate, a description of the study was provided to students via an announcement during class and also an information letter. In addition, parents were sent a letter that informed them of the purpose of the study and provided them with the opportunity to decline participation (passive parental consent procedures). Two weeks after letters were provided to both adolescents and parents, adolescents were invited to complete a questionnaire package during a pre-arranged class. Adolescent consent was denoted by adolescents electing to complete the questionnaire.

### Measures

#### Transformational parenting

Adolescents’ perceptions of parents’ transformational behaviors were assessed using the Transformational Parenting Questionnaire [TPQ; [26]. In the present study, adolescents completed either one (single parent family) or two (dual parent family) separate TPQ’s for their family. The 16-item TPQ contains separate subscales designed to measure the four dimensions of transformational parenting, with four items per subscale. Items on the TPQ are prefixed with the stem “My parent/guardian…” with exemplar items including “acts as a person that I look up to” (idealized influence), “Is optimistic about what I can accomplish” (inspirational motivation), “Gets me to think for myself” (intellectual stimulation), and “Displays a genuine interest in my life” (individualized consideration). Responses are anchored on a six-point rating scale from 0 (strongly disagree) to 5 (strongly agree). Morton and colleagues [26] provided evidence for construct validity of measures derived from the TPQ, with the most parsimonious operationalization represented by a higher-order dimension of “transformational parenting”, that is measured by scores derived from the four transformational parenting subscales. In the present study, the subscales were summed to yield total transformational parenting scores between 0 and 80, where higher scores suggest a higher level of perceived transformational parenting behaviors. This higher-order measure of transformational parenting was used, and demonstrated acceptable internal consistency for both mothers’ (Cronbach α = .95) and fathers’ scores (Cronbach α = .96).

#### Dietary behaviors

Adolescents’ dietary behaviors were assessed using the Adolescent Food Habits Checklist [AFHC; [35]. This instrument was designed specifically for adolescents and requires participants to complete 23 items pertaining to fat and fibre (i.e., “I usually avoid eating fried foods”), simple sugars (i.e., “I often buy pastries or cakes”), and fruit and vegetable intake (i.e., “I usually eat at least one serving of vegetables (excluding potatoes) or salad with my evening meal”). Items were scored as true, false, or not applicable. In order to calculate a dietary behavior score [cf. [35], all items representing healthy food choices and behaviors were given a value of 1 and the final score was adjusted for the “not applicable” and missing responses using the formula: AFHC = number of healthy responses x (23/number of items completed). Previous research with this instrument has demonstrated acceptable internal reliability (Cronbach’s α = 0.82) and evidence for convergent validity on measures derived from the AFHC in relation to other measures of dietary fat intake (r = −.46), daily fruit and vegetable intake (r = .45), as well as dietary restraint (r = .17) with adolescents [35].

#### Leisure time physical activity

Leisure-time physical activity was measured using the Godin Leisure-time Exercise Questionnaire [LTEQ; [36]. This instrument requires participants to report the number of times they participate in strenuous, moderate and light exercise during a typical week (for more than 15 minutes). A total score was calculated by multiplying the weekly frequencies of strenuous, moderate, and light activities by 9, 5, and 3, respectively, for a total metabolic-equivalent intensity value. This instrument has been used with adolescents and demonstrated adequate test-retest reliability coefficients (.69 < r < .96), and acceptable evidence for concurrent validity through significant correlations with other self-report instruments (r = .32). In addition, measures derived from this instrument have been shown to have acceptable criterion-related validity (r = .36) with accelerometry measures [37].

### Data analysis

#### Preliminary analyses

Prior to analyses, data were screened for missing values, accuracy of data entry, outliers and normality. In terms of normality, all the skewness values ranged from .28 to 1.71 and the kurtosis values ranged from .32 to 5.03 for all variables which indicates similarity to the normal curve [38]. Examination of the assumptions associated with regression analyses (i.e., normality, linearity and homoscedasticity) suggested that there were no problems in the data. Finally, the Durbin–Watson statistic was employed as a diagnostic check for bias resulting from correlated errors terms. These values were in the recommended range (1.79–1.89) for all reported equations [39]. As some adolescents had missing data for some of the variables (<5% missing data), the number differed slightly between different analyses, as indicated below (only complete cases were utilized in the analysis).

In the majority of cases, the family unit is comprised of two parents (a mother and a father). Consistent with other family research [40,41] adolescents’ perceptions of the leadership behaviors of both parents was averaged, to take into account any compensating effects that may exist if one parent is perceived as highly transformational and the other parent is perceived as low in transformational behaviors. In families where only one parent was specified (i.e., single parent families) compensating effects do not take place and, as such, in these instances a single parenting score was utilized. This score is reflective of the *family unit* (regardless of whether the family is comprised of one or two parents), and represented the ‘family transformational leadership’ as perceived by the adolescent. This score was used in the subsequent regression analyses. Thirty-two adolescents completed the TPQ with reference to a guardian other than a parent (i.e., aunt, uncle, grandmother, grandfather). These cases were excluded from the analyses (these included cases where two TPQ’s were completed for a biological parent *and* another relative). Descriptive statistics, including unadjusted means, standard deviations, and intercorrelations between the study variables are presented in Table 1.

**Table 1:** Unadjusted means, standard deviations, and intercorrelations for the study variables

	**M**	**SD**	**1**	**2**	**3**
1. Family Transformational Leadership	60.280	13.705	--	.271*	.141*
2. Adolescent Healthy Eating Behavior	12.911	4.878		--	.025
3. Adolescent Physical Activity	55.903	32.567			--

*Notes: n* = 822. Family transformational parenting scores range from 0–80. Healthy eating scores range from 0 to 23. Physical activity score ranges from 0–225. *M* = unadjusted means. SD = standard deviations * *p* < .01.

#### Regression analysis

Two separate hierarchical regression analyses were performed on the entire data set (*n* = 822 for dietary behaviors and *n* = 798 for physical activity behaviors) with ‘family transformational leadership’ specified as the independent variable and adolescent health behaviors (dietary and physical activity) as the dependent variables. In order to control for the influence of key demographic variables on dietary and physical activity behaviors, child gender (1 = Male, 2 = Female), child ethnicity (1 = Caucasian, 2 = Non-Caucasian) and family structure (1 = Single-parent family, 2 = Dual-parent family) were first entered into the analyses for each regression analyses (step 1). Following this, the ‘family transformational leadership’ score was entered in order to determine the relationship between adolescent perceptions of transformational parenting behaviors and adolescent health behaviors (step 2).

#### Extreme group comparisons

A further examination of the relationships between family transformational leadership and adolescent health behaviors was conducted via extreme group comparisons. An extreme group approach (EGA) is a useful strategy in exploratory analyses as this approach increases statistical power and enhances the detection of general trends that might be overlooked with the inclusion of a full range of data [42]. Using a quartile split procedure on the entire data set (*n* = 825), parents were classified as ‘high transformational’ (i.e., parent scored above 75^th^ percentile on TPQ) or ‘low transformational’ (i.e., parent scored below 25^th^ percentile on TPQ). A HIGH TP family consisted of both the mother and father scoring above the 75^th^ percentile, a LOW TP family consisted of both the mother and father scoring in the 25^th^ percentile, and a HIGH-LOW TP family consisted of one parent scoring above the 75^th^ percentile and the other parent scoring in the 25^th^ percentile (in the case of single parent families, the one parent for which data was provided was also considered and utilized in the analysis; i.e., single parent scores above 75th percentile were grouped in the HIGH TP group and single parent scores within the 25th percentile were grouped in the LOW TP group). In Table 2, adolescent dietary and physical activity behavior means (and standard deviations) of the three extreme groups (HIGH TP family, LOW TP family and HIGH-LOW TP family) are presented.

**Table 2:** Comparisons of high transformational, low transformational and high-low transformational families

	**High Transformational Parenting Families (HIGH TP family)**		**Low Transformational Parenting Families (LOW TP family)**		**One High-One Low Transformational Parenting Families(HIGH-LOW TP family)**		**η2**
	M	SD	M	SD	M	SD	
Healthy Eating	14.642	4.633	11.331	4.966	11.386	3.978	.106*
Physical Activity	59.315	27.917	48.467	29.245	57.648	42.376	.031*

*Notes: n* = 137 for HIGH TP families, *n* = 165 for LOW TP families and *n* = 26 for HIGH-LOW TP families. Healthy eating scores range from 0 to 23. Physical activity score ranges from 0–225. For η2, .*01* corresponds to a *small* effect, .*06* to a *medium* effect and .*14* to a *large* effect [38].

*M* = adjusted means. SD = standard deviations. η2 = eta squared. * *p* < .01.

First, three one-way analysis of variance (ANOVA) were computed to examine whether the three groups differed in terms of key demographic variables. This indicated that there were no differences in ethnicity between the three groups (*F* (2, 336) =1.954, *p* = .143). However, the three groups were different in terms of gender (*F* (2, 334) =3.951, *p* = .020), with more female adolescents reported in the HIGH TP group than the LOW TP group (*p* = .020). There were no differences between the HIGH-LOW TP group and either the HIGH or LOW TP groups with regards to adolescent gender (*p* = .524) With regards to family structure (single versus dual parents), there were no differences between the HIGH and LOW TP groups (*p* = .103)^2^. Therefore, in the subsequent analyses only ‘gender’ was specified as a covariate. A multivariate analysis of covariance (MANCOVA) was performed in order to examine differences in adolescent health behaviors (while controlling for child gender) that exist between (a) families whereby the *family unit* was perceived by the adolescent to display (consistent) high levels of transformational behaviors (i.e., both parents above the 75^th^ percentile for transformational parenting; HIGH TP family), (b) families whereby the family unit was perceived by the adolescent to display (consistent) low levels of transformational behaviors (i.e., both parents below the 25^th^ percentile; LOW TP family), and (c) families in which parents were perceived to display inconsistent transformational behaviors (i.e., one parent perceived as displaying HIGH transformational behaviors and one parent perceived as displaying LOW transformational parenting; HIGH-LOW TP family). Follow up univariate *F* tests were computed to examine differences between groups for dietary and physical activity behaviors.

## Results

### Regression analyses

#### Dietary behaviors

Results of the hierarchical regression analysis indicated that step 1 was significant (*F* (3,818) =9.129, *p* < .001, *R*_adjusted_^2^ = .029). Gender contributed significantly to dietary behaviors (*b* =1.603, (95% CI =0.945, 2.262), *p* < .001) in the direction that female adolescents reported more healthy dietary behaviors than male adolescents. Family structure also contributed to dietary behaviors (*b* = .821, (95% CI =0.051, 1.590), *p* = .037) in the direction that adolescents from two parent families reported more healthy eating behaviors than did adolescents from single parent families. Ethnicity did not contribute to dietary behaviors (*b* = −.207, (95% CI = −1.00, 0.586), *p* = .609). After controlling for the effects of gender, family structure and ethnicity in step 1, family transformational parenting contributed significantly to dietary behaviors (*F* (1,817) =56.56, *p* < .001, *R*_adjusted_^2^ = .090, Δ*R*^2^ = .062. Adolescent perceptions’ of higher family transformational parenting scores predicted more healthy eating behaviors (*b* = 0.090, (95% CI = 0.066, 0.113), *p* < .001).

#### Physical activity

Results of the hierarchical regression analysis indicated that step 1 was significant (*F* (3,794) =12.603, *p* < .001, *R*_adjusted_^2^ = .042). Gender contributed significantly to physical activity behaviors (*b* = −8.634, (95% CI = −13.068, -4.200), *p* < .001) in the direction that males reported greater leisure time physical activity than females. Ethnicity also contributed to physical activity (*b* = −12.089, (95% CI = −17.414, -6.764), *p* < .001) in the direction that Caucasian adolescents reported more physical activity behaviors than non-Caucasian adolescents. Family structure did not contribute to physical activity (*b* = −3.791, (95% CI = −9.002, 1.421), *p* = .154). After controlling for the effects of gender, family structure and ethnicity in step 1, family transformational parenting contributed significantly to leisure-time physical activity behaviors (*F* (1,793) =20.195, *p* < .001, *R*_adjusted_^2^ = .064, Δ*R*^2^ = .024). Adolescent perceptions’ of higher family transformational parenting scores predicted greater leisure time physical activity behaviors (*b* = 0.369, (95% CI = 0.208, 0.503), *p* < .001).

### Extreme group comparisons

The omnibus MANCOVA revealed that the three groups differed significantly on adolescent health behaviors (*F* (4, 646) = 12.158, Wilks’ λ = .865, *p* < .001, η^2^ = .070) after controlling for adolescent gender. Given the significance of the overall MANCOVA, the univariate main effects were examined. Significant univariate main effects were found for both adolescents’ dietary (*F* (2, 324) =19.128, *p* < .001, η^2^ = .106) and physical activity behaviors (*F* (2, 324) = 5.208, *p* = .006, η^2^ = .031). Univariate comparisons of means, standard deviations and effect sizes for each of the three groups are shown in Table 2. For adolescent dietary behaviors, significant pairwise comparisons (*p* < .001) were obtained between the HIGH TP family group (*M* adolescent healthy eating score = 14.642) and the LOW TP family group (*M* = 11.331). Significant pairwise comparisons (*p* = .001) were also obtained between the HIGH TP family group (*M* = 14.642) and inconsistent HIGH-LOW TP family group (*M* = 11.386). No significant differences were observed (*p* = .956) between the LOW TP family group (*M* = 11.331) and inconsistent HIGH-LOW TP family group (*M* = 11.386). For adolescent physical activity behaviors, significant pairwise comparisons were obtained (*p* = .002) between HIGH TP family group (*M* adolescent leisure time physical activity score = 59.315) and LOW TP family group (*M* = 48.467). No significant differences were observed between inconsistent HIGH-LOW TP family (*M* = 57.648) and the HIGH TP family group (*M* = 59.315, *p* = .791) or the LOW TP family group (*M* = 48.467, *p* = .141) although the means were in the hypothesized direction.

## Discussion

The results of this cross-sectional study revealed that family transformational parenting behaviors were positively associated with both healthful dietary intake and leisure-time physical activity levels amongst adolescents. This suggests that family leadership processes may be important factors for adolescent health-enhancing behaviors, and lends support for the utility of transformational leadership theory as a viable framework for understanding the role of parents in relation to adolescent health behaviors. Given that dietary and exercise habits during adolescence contribute towards a reduced risk of health problems that extend across the lifespan [43,44], these findings provide some indication that the family context is particularly salient for the socialisation of these behaviors, and supports research in that suggests parents’ influence over adolescents does not diminish as children mature into adolescents [32].

Previous research has examined the relationship between adolescents’ perceptions of transformational parenting behaviors as displayed by mothers’ and fathers’ in relation to self-regulatory efficacy beliefs for physical activity and healthy eating as well as life satisfaction [26]. The present study extends this line of research by examining actual health *behaviors* and found that perceptions of transformational parenting behaviors exhibited within the *family unit* were associated with both healthy dietary intake and leisure-time physical activity behaviors.

For adolescent dietary behaviors, the results of the present study indicate that within a family unit, inconsistent levels of transformational parenting (i.e., one parent above the 75^th^ percentile and one parent within the 25^th^ percentile for transformational parenting behaviors) were associated with the same levels of adolescent healthy eating as those families whose parents lay within the 25^th^ percentile. This suggests that to bring about improved adolescent dietary behaviors, the family unit (regardless of single or dual parent families) need to be perceived as displaying relatively high levels of transformational parenting behaviors by their adolescent children. Furthermore, in the case of dual parent families, both parents need to be on the same page when it comes to transformational parenting approaches (i.e., both parents should display high levels of these behaviors).

For physical activity behaviors, although adolescents from families displaying consistently high levels of transformational parenting reported greater levels of physical activity than adolescents from families displaying consistently low levels of transformational parenting, adolescents reporting inconsistent parenting did not report different levels of physical activity than adolescents from either the HIGH or LOW TP families. Although the effects of inconsistent transformational parenting for adolescent physical activity are unclear, the findings lend further support to the fact that adolescents who perceive the family unit (regardless of family structure) to display consistent and high levels of transformational parenting tend to display greater health-enhancing behaviors than adolescent from families who are perceived as displaying low levels of transformational parenting behaviors.

In spite of the potential contributions of this study, there are several limitations that must be noted. First, the small number of families in the HIGH-LOW TP group did not permit us to investigate whether the effects of extreme inconsistency of transformational parenting behaviors varies depending on whether the highly transformational parent is male or female. This reflects the fact that parenting behaviors are generally highly correlated for mothers and fathers [33,34]. However, this remains an important question for future research.

Another limitation reflects the research design. This research was cross-sectional in nature, which not only limits potential inferences of causality, but also increases the possibility of common method variance across participants’ predictor and criterion responses. With respect to this latter point, however, it should also be noted that a different response format was used in the assessment of the predictor and criterion measures, which has been shown to mitigate common method bias [45]. In future, the hypotheses tested in this study should be examined through use of a longitudinal design, ideally with objective measures of adolescent dietary and physical activity behaviors. Furthermore, studies in the organizational domain have demonstrated the efficacy of short-term interventions to develop and enhance transformational leadership behaviors [30]. Research surrounding whether transformational leadership behaviors in families can be developed through intervention represents a fruitful area of research in order to support families in their parenting roles to bring about positive changes in adolescent health.

Finally, the variance in adolescent health behaviors explained by family transformational leadership was relatively low (i.e., *R*_adjusted_^2^ < 10%). However, it is important to note that even small amounts of variance explained can prove important [46]. For example, even a small improvement in adolescent dietary and physical activity behaviors could translate into a significant public health impact [47]. Therefore, if the efficacy of parenting interventions guided by the tenets of transformational leadership theory can be established, the implications for improved adolescent health behaviors are far-reaching.

Although mediating relationships were not examined in this exploratory study, it seems plausible to suggest that adolescents from families displaying high levels of transformational behaviors are more likely to feel confident and empowered to achieve a higher level of functioning and pursue voluntary health behaviors [9,26]. This is consistent with research in other domains that suggests transformational leadership predicts both follower self-efficacy [27] and self-regulation [48] and pro-active behaviors [49]. Future research should seek to address mediating pathways by exploring intra-personal variables such as adolescent self-efficacy, self-determined motivation, self-esteem and health-related attitudes [9]. Furthermore, future research should seek to address potential moderators of the transformational parenting – adolescent health behavior relationship, such as parents own’ health behaviors, their contact time with their children, and socio-economic status [9]. Finally, future studies should seek to establish evidence of generalizability (i.e., the extent to which the effects of transformational leadership within families can generalize across different populations, such as younger children or older adolescents and young adults). This will ultimately help to provide further support for the transformational parenting construct and build upon the evidence presented here in order to develop a conceptual framework to develop future family based interventions targeting family leadership behaviors.

## Conclusions

This paper represents the first empirical study to explore family transformational leadership behaviors in relation to adolescent health-enhancing behaviors, and suggests that transformational leadership theory is a useful framework for understanding and potentially fostering adolescent healthy eating and physical activity behaviors. If future experimental research can demonstrate success in fostering transformational leadership in families, this framework holds potential for improving adolescent dietary and physical activity practices via improvements in family leadership processes.

## Endnotes

1. The data presented in this paper represent part of a larger program of research designed to examine transformational parenting behaviors in relation to various adolescent health outcomes. Data on the reliability and factorial validity of measures derived from the Transformational Parenting Questionnaire (TPQ) were previously published in Morton et al. (2011). Data on physical activity and dietary behaviors reported in this manuscript were not presented in the paper by Morton et al. (2011).

2. Given that the HIGH-LOW TP group contained solely dual-parents, we were only interested in determining whether there were differences in family structure (single versus dual parent families) between the HIGH TP and LOW TP groups.

## Competing interests

The authors declare that they have no competing interests.

## Authors' contributions

KM and MB conceptualized and designed the study. KM collected the data. KM, AW and LP contributed to the statistical analyses. KM, AW, LP and MB contributed to the writing of the manuscript. All authors read and approved the final manuscript.
